# 

**Published:** 1897-11

**Authors:** 


### BOOKS RECEIVED.

A Text-Book of the Practice of Medicine. By James M. Anders, M. D., Ph. D., LL. D., Professor of the Practice of Medicine and of Clinical Medicine in the Medico-Chirurgical College, Philadelphia;

Attending Physician to the Medico-Chirurgical and Samaritan Hospitals, Philadelphia, etc. Octavo, pp. 1287. Illustrated. Price, cloth, $5.50 net; sheep or one-half morocco, $6.50 net. Philadelphia: W. B. Saunders, 925 Walnut street. 1896.

A Manual of Medical Jurisprudence. By Alfred S. Taylor, M. D., Lecturer on Medical Jurisprudence and Chemistry in Guy's Hospital, London. New American edition of 1897 from the twelfth English edition. Thoroughly revised by Clark Bell, Esq., of the New York Bar. In one octavo volume of 831 pages, with fifty-four engravings and eight full-page plates. Cloth, $4.50; leather, $5.50. Philadelphia and New York: Lea Brothers & Co., Publishers. 1897.

Cutaneous Medicine. A systematic treatise on the diseases of the skin. By Louis A. Duhring, M. D., Professor of Diseases of the Skin in the University of Pennsylvania, etc. Octavo, pp. 223-494. Part II. Classification; anemias; hyperemias; inflammations. Illustrated. Philadelphia: J. B. Lippincott Co. 1898.

Pathological Technique. A practical manual for the pathological laboratory. By Frank B. Mallory, A. M., M. D., Assistant Professor of Pathology, Harvard University Medical School; Assistant Pathologist to the Boston City Hospital; Pathologist to the Children's Hospital and the Carney Hospital, Boston, and James H. Wright, A. M., M. D., Director of the Laboratory of the Massachusetts General Hospital; Instructor in Pathology, Harvard University Medical School. Octavo, 397 pages, with 105 illustrations. Price, cloth, $2.50 net. Philadelphia: W. B. Saunders, 925 Walnut street. 1897.

Traumatic Injuries of the Brain and Its Membranes. With a special study of pistol-shot wounds of the head in their medico-legal and surgical relations. By Charles Phelps, M. D., Surgeon to Bellevue and St. Vincent's Hospitals. Octavo, pp. xiv.—582. With forty-nine illustrations. New York: D. Appleton & Co. 1897.

A Manual of Legal Medicine for the use of Practitioners and Students of Medicine and Law. By Justin Herold, A. M., M. D., formerly Coroner's Physician of New York City and County, etc., etc. Octavo, pp. xv.—678. Philadelphia: J. B. Lippincott Co. 1898.

Lectures on the Malarial Fevers. By William Sidney Thayer, M. D., Associate Professor of Medicine in the Johns Hopkins University. Small octavo, pp. vi.—326. New York: D. Appleton & Co. 1897.

Essentials of Bacteriology: being a concise and systematic introduction to the study of microörganisms, for the use of students and practitioners. By M. V. Ball, M. D., Bacteriologist to St. Agnes' Hospital, Philadelphia. Duodecimo, pp. xvi.—218. Third edition, revised. Illustrated. Price, $1.00. Philadelphia: W. B. Saunders, 925 Walnut street.

A Practical Treatise on Sexual Disorders of the Male and Female. By Robert W. Taylor, M. D., Clinical Professor of Venereal Diseases in the College of Physicians and Surgeons, New York. In one handsome octavo volume of 448 pages, with seventy-three illustrations and eight plates in color and monochrome. Cloth, $ 3.00 net. New York and Philadelphia: Lea Brothers & Co.

The Essentials of Obstetrics. By Charles Jewett, M. D., Professor of Obstetrics in the Long Island College Hospital, Brooklyn, New York.

In one handsome 12mo volume of 356 pages, with seventy-eight illustrations and three colored plates. Cloth, $2.25. New York and Philadelphia. 1897.

Incompatibilities in Prescriptions. For students in pharmacy and medicine and practising pharmacists and physicians. By Edsel A. Ruddiman, Ph. M., M. D., Adjunct Professor of Pharmacy and Materia Medica in Vanderbilt University. Octavo, pp. 267. Price, $2.00. New York: John Wiley & Sons; London: Chapman & Hall, Limited. 1897.

